# Supplementary material for: Single rate-limiting event of carcinogenesis
Source: Nat Commun. 2025 Oct 30;16:9596. doi: 10.1038/s41467-025-64613-6 (PMC12575674; doi:10.1038/s41467-025-64613-6)
Supplement: Supplementary file 1 — Supplementary Information [file 41467_2025_64613_MOESM1_ESM.pdf]

Supplementary Note 1

Variation of individual-level risk within and between subpopulations

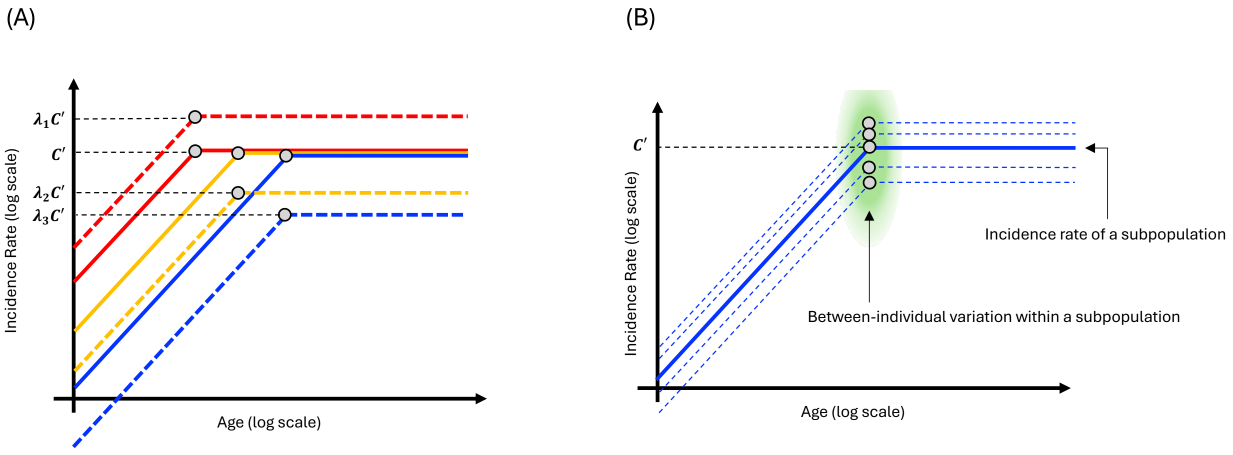

Figure S1. Variation of constant incidence between subpopulations and within a subpopulation. (A) Two sets of subpopulation composition that produce the same population-level incidence. The set of subpopulations represented by dotted red, yellow, and blue lines have the constant incidence rates that are λ_1, λ_2, and λ_3 times, respectively, of the constant incidence C' that is shared by the solid-line set of subpopulations. If the subpopulation proportions in the dotted-line set are $1/\lambda_1$, $1/\lambda_2$, and $1/\lambda_3$ times of the subpopulation proportions of their same-colored counterparts in the solid-line set, then the dotted-line set and the solid-line set have the same population-level incidence by age. (B) Variation in age-specific incidence rates across individuals in a single subpopulation. The solid line represents the mean incidence by age of a subpopulation that is comprised of individuals who transition at the same age, where the individuals' propensity level varies due to between-individual variation of risk factors.

The Postulate model (1) warrants further discussion as it is undoubtedly a simplified model of individual-level cancer incidence. First, as Figure S1 Panel (A) demonstrates together with Figure 4 Panels (A) vs. (B), the same population-level incidence can be represented equally well by numerous sets of subpopulations if the ages at the phase transition are shared among the sets and the subpopulation proportions are inverse-proportionally adjusted according to the levels of the constant incidence rates. Thus, the constant incidence value shared across subpopulations in the Postulate model (1) does not have to be a single common value across subpopulations. Had we allowed the constant incidence value to vary by propensity of subpopulations in the Postulate model (1), we would have arrived at the same goodness of fit, but with different subpopulation proportions. Recall, however, that the constant incidence rate in the contralateral breast following the first breast cancer[9] was the same irrespective of the age at the first breast

cancer.[11,12] We observed the same feature in renal-cell carcinoma here (Figure 1). Thus, the empirical evidence supports the solid-line sets of Figure S1 Panel (A) with the common constant incidence rate, and the conclusions above regarding the subpopulation proportions (i.e., those on selective pressure and early-life risk of certain cancer types) hold. The 1.0% per year value we used for the constant incidence is an approximate value taken from breast cancer data,[11,12] but it could differ by cancer type.

Second, between-individual variation of the incidence must exist within a subpopulation due to between-individual variation of risk factors. Contralateral breast cancer incidence rates are associated with both genetic and environmental/lifestyle risk factors.[26,27] These observations are consistent with the Postulate framework, in which heterogeneity in propensity reflects the combined influence of multiple genetic and environmental/lifestyle determinants. As Figure S1 Panel (B) illustrates, the subpopulation incidence is the mean incidence of individuals in the subpopulation with a common phase-transition age: small-scale variation in their propensity level and constant incidence rate around the means can exist due to between-individual variation of risk factors. For example, smoking habits[23] and HPV infection patterns[24] are major risk factors of lung cancer and cervical cancer, respectively, perhaps with between-individual variation within families that influence the propensity: withdrawal of these major risk factors such as smoking cessation would significantly alter the propensity and the power-law increase of the number of precancer cells associated with the exposure. Between-individual heterogeneity in propensity, as represented in the Postulate model (1), extends beyond the discrete subpopulations used for modeling convenience here and exists on a continuum.

Supplementary Note 2

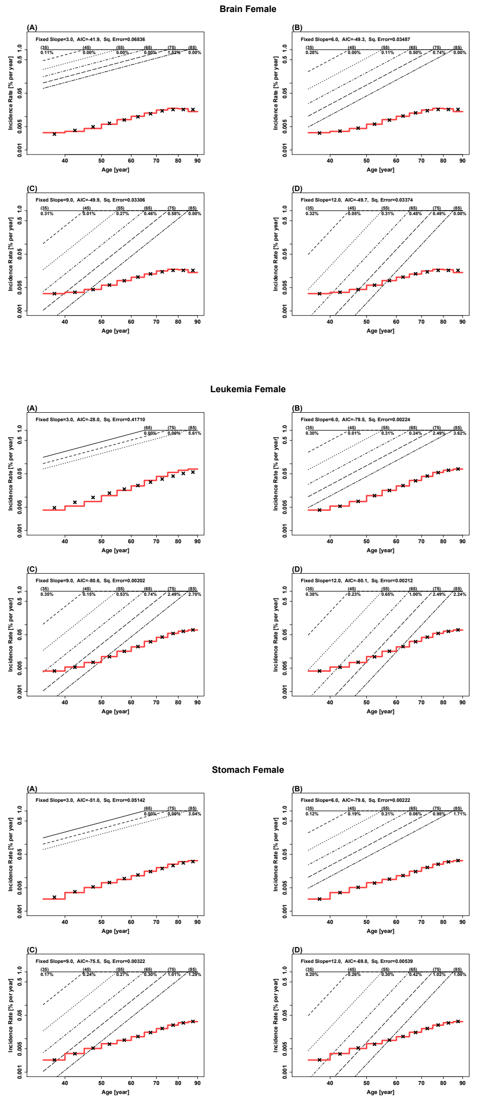

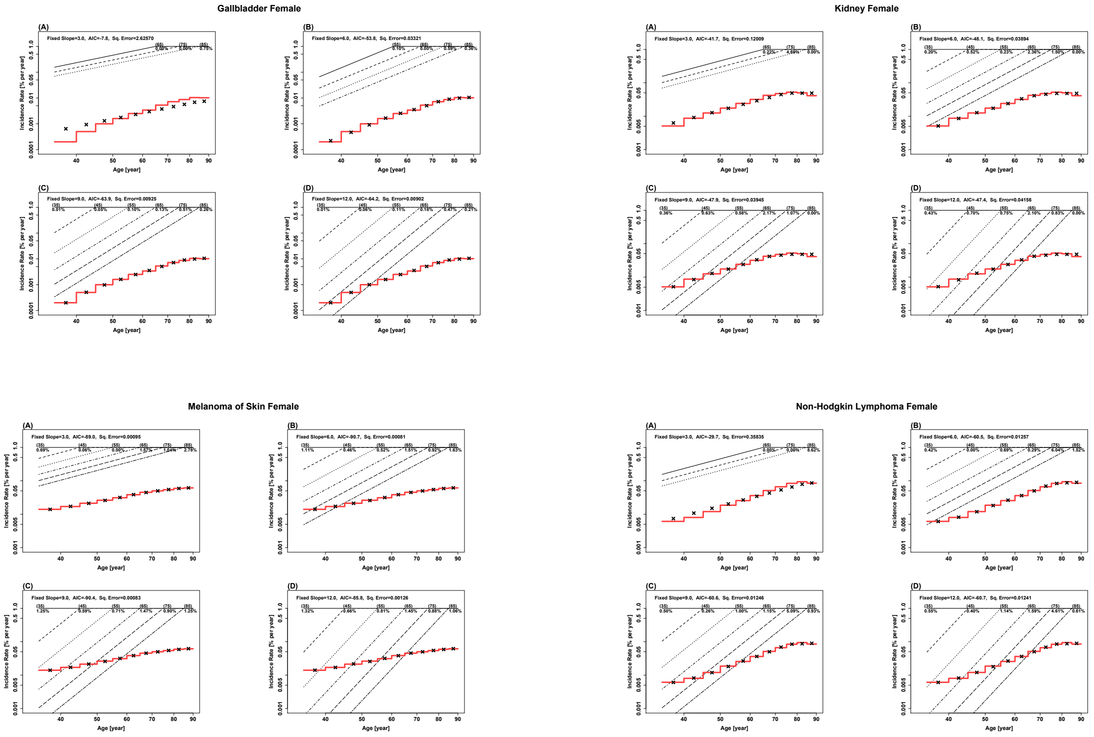

Figure S2. Fits of Peto-Mack Model with up to six subpopulations with distinct propensity levels to the observed population-level age-specific incidence rates in 5-year age groups of various cancer types in females from the Surveillance, Epidemiology, and End Results program. All panels are in the log-log scale. Panels (A), (B), (C), and (D) considered $k = 4$, 7, 10, and 13, respectively. The high constant rate of the Model was set at 1.0%/year in all four panels. In each analysis, the subpopulation size, m, of 6, 5, 4, and 3 were considered where m=6 subpopulations are those reaching at the high constant rate at age 35, 45, 55, 65, 75, and 85, and m<6 subpopulations would remove the first (6-m) of these subpopulations. The fits were obtained by least square methods of log rates and the best subpopulation size, m, was selected by Akaike's Information Criterion (AIC).

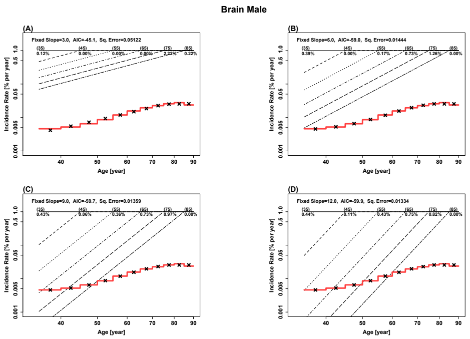

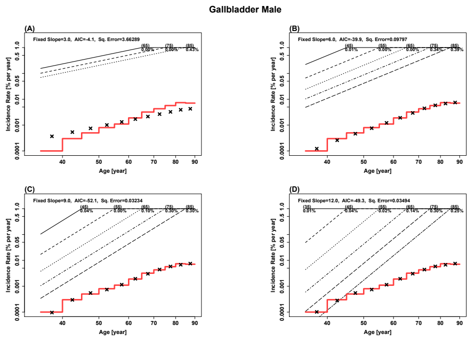

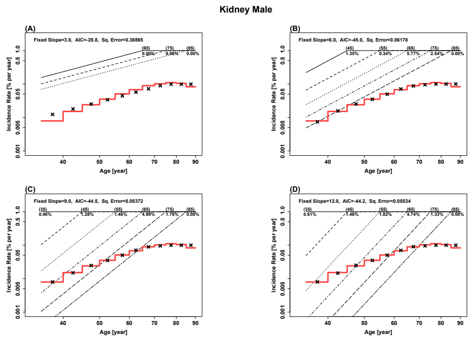

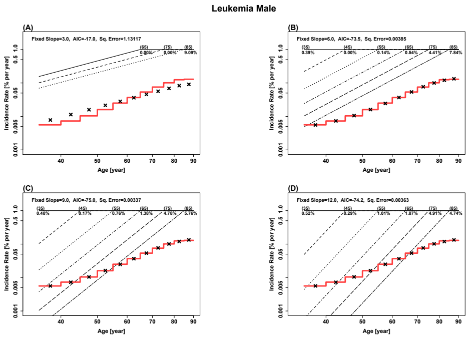

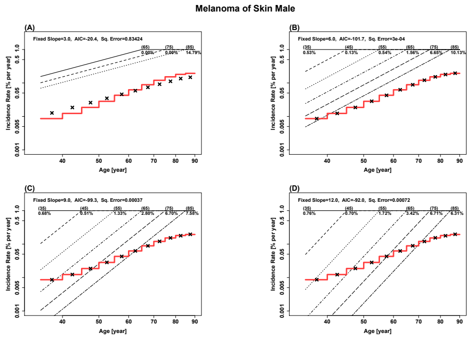

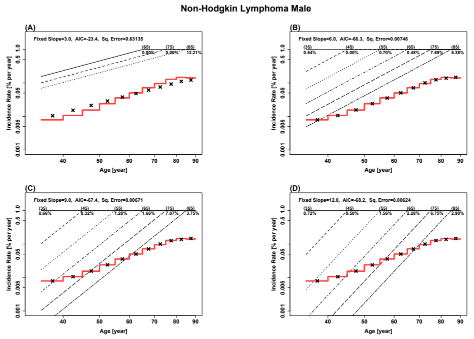

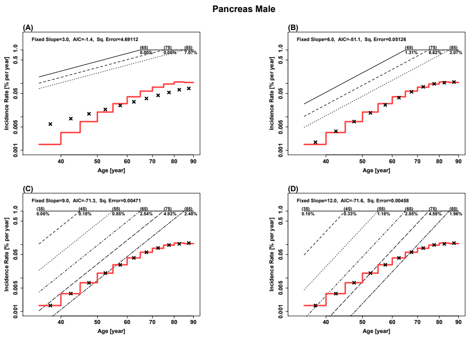

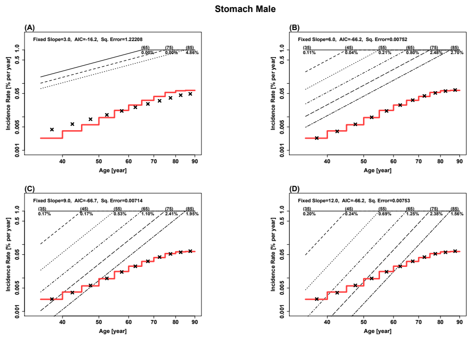

Figure S3. Fits of Peto-Mack Model with up to six subpopulations with distinct propensity levels to the observed population-level age-specific incidence rates in 5-year age groups of various cancer types in males from the Surveillance, Epidemiology, and End Results program. All panels are in the log-log scale. Panels (A), (B), (C), and (D) considered $k = 4$, 7, 10, and 13, respectively. The high constant rate of the Model was set at 1.0%/year in all four panels. In each analysis, the subpopulation size, *m*, of 6, 5, 4, and 3 were considered where *m*=6 subpopulations are those reaching at the high constant rate at age 35, 45, 55, 65, 75, and 85, and *m*<6 subpopulations would remove the first (6-*m*) of these subpopulations. The fits were obtained by least square methods of log rates and the best subpopulation size, *m*, was selected by Akaike's Information Criterion (AIC).

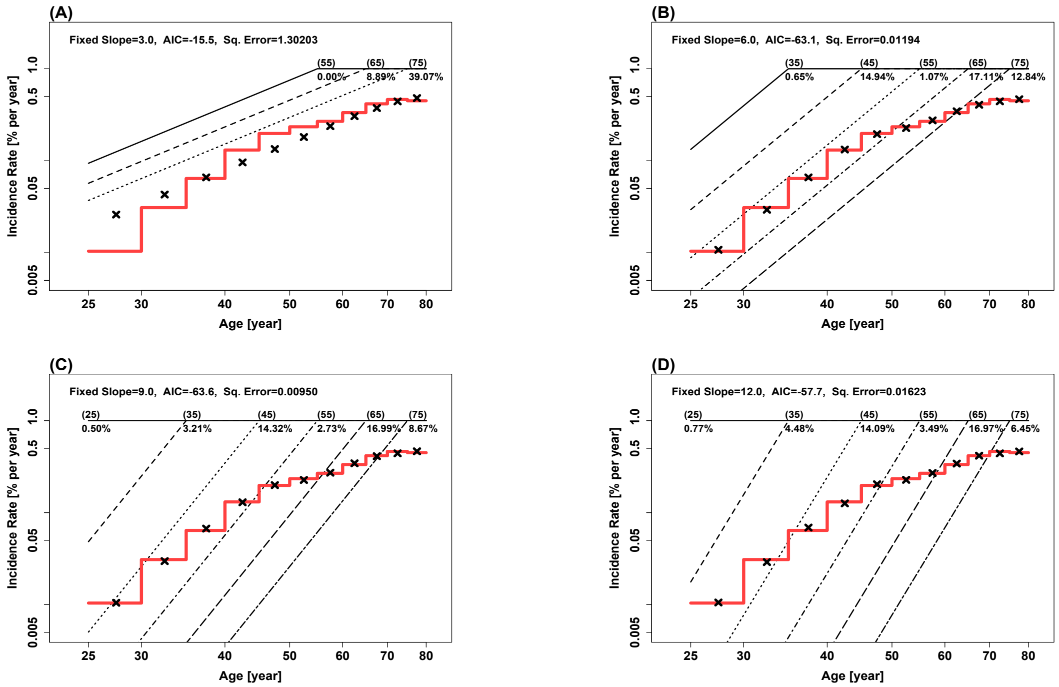

Figure S4. Fits of Peto-Mack Model with up to six subpopulations with distinct propensity levels to the observed population-level age-specific incidence rates in 5-year age groups of breast cancer in females from the Surveillance, Epidemiology, and End Results program. All panels are in the log-log scale. Panels (A), (B), (C), and (D) considered $k = 4, 7, 10,$ and 13, respectively. The high constant rate of the Model was set at 1.0%/year in all four panels. In each analysis, the subpopulation size, m, of 6, 5, 4, and 3 were considered where m=6 subpopulations are those reaching at the high constant rate at age 25, 35, 45, 55, 65, and 75, and m<6 subpopulations would remove the first (6-m) of these subpopulations. The fits were obtained by least square methods of log rates and the best subpopulation size, m, was selected by Akaike's Information Criterion (AIC). The 11 5-year age groups started at 25-29 years for female breast cancer due to high incidence from 25-29 years old. The best fit models were obtained with $k = 7$ and $k = 10$, with the latter with 6 subpopulations providing a slightly better fit. The general features we observed in the Model fits for other cancer types above are also seen for female breast cancer. There are, however, two unique features in the breast cancer analysis results. One is that the size of the subpopulation that reaches the high constant rate at age 55 is less than 3% of the population, while the subpopulations that reach the high constant rate before (at age 45) and after (at age 65) are both much larger, each greater than 14% of the population. This indicates the influence of menopause on the incidence rate of female breast cancer around the menopausal age. The other unique feature is that nearly half of the population is in the subpopulations that reach the high constant rate at age 75 or earlier, a much higher proportion than the corresponding proportion for the other cancer types we studied.
