# Supplementary material for: Single rate-limiting event of carcinogenesis
Source: Nat Commun. 2025 Oct 30;16:9596. doi: 10.1038/s41467-025-64613-6 (PMC12575674; doi:10.1038/s41467-025-64613-6)
Supplement: Supplementary file 3 — Reporting Summary [file 41467_2025_64613_MOESM3_ESM.pdf]

nature portfolio

Corresponding author(s): Yutaka Yasui

Last updated by author(s): Sep 10, 2025

Reporting Summary

Nature Portfolio wishes to improve the reproducibility of the work that we publish. This form provides structure for consistency and transparency in reporting. For further information on Nature Portfolio policies, see our Editorial Policies and the Editorial Policy Checklist.

Statistics

For all statistical analyses, confirm that the following items are present in the figure legend, table legend, main text, or Methods section.

n/a	Confirmed	
☐	☒	The exact sample size (*n*) for each experimental group/condition, given as a discrete number and unit of measurement
☒	☐	A statement on whether measurements were taken from distinct samples or whether the same sample was measured repeatedly
☒	☐	The statistical test(s) used AND whether they are one- or two-sided<br>*Only common tests should be described solely by name; describe more complex techniques in the Methods section.*
☒	☐	A description of all covariates tested
☐	☒	A description of any assumptions or corrections, such as tests of normality and adjustment for multiple comparisons
☐	☒	A full description of the statistical parameters including central tendency (e.g. means) or other basic estimates (e.g. regression coefficient) AND variation (e.g. standard deviation) or associated estimates of uncertainty (e.g. confidence intervals)
☒	☐	For null hypothesis testing, the test statistic (e.g. *F*, *t*, *r*) with confidence intervals, effect sizes, degrees of freedom and *P* value noted<br>*Give P values as exact values whenever suitable.*
☒	☐	For Bayesian analysis, information on the choice of priors and Markov chain Monte Carlo settings
☒	☐	For hierarchical and complex designs, identification of the appropriate level for tests and full reporting of outcomes
☒	☐	Estimates of effect sizes (e.g. Cohen's *d*, Pearson's *r*), indicating how they were calculated

Our web collection on statistics for biologists contains articles on many of the points above.

Software and code

Policy information about availability of computer code

Data collection	None
Data analysis	R statistical software. R Core Team (2021). R: A language and environment for statistical computing. R Foundation for Statistical Computing, Vienna, Austria. (https://www.r-project.org/).

For manuscripts utilizing custom algorithms or software that are central to the research but not yet described in published literature, software must be made available to editors and reviewers. We strongly encourage code deposition in a community repository (e.g. GitHub). See the Nature Portfolio guidelines for submitting code & software for further information.

Data

Policy information about availability of data

All manuscripts must include a data availability statement. This statement should provide the following information, where applicable:

- Accession codes, unique identifiers, or web links for publicly available datasets
- A description of any restrictions on data availability
- For clinical datasets or third party data, please ensure that the statement adheres to our policy

All data used in the manuscript are available from Reference 4 and 32.
4. SEER*Explorer: An interactive website for SEER cancer statistics. Surveillance Research Program, National Cancer Institute; 2023 Apr 19. SEER Incidence Data, November 2022 Submission (1975-2020), SEER 22 registries. (https://seer.cancer.gov/statistics-network/explorer/).

32. Surveillance, Epidemiology, and End Results (SEER) Program (www.seer.cancer.gov) SEER*Stat Database: Incidence - SEER Research Data, 8 &17 Registries, Nov 2023 Sub (2000-2021) - Linked To County Attributes - Time Dependent (1990-2022) Income/Rurality, 1969-2022 Counties, National Cancer Institute, DCCPS, Surveillance Research Program, released April 2024, based on the November 2023 submission.

Research involving human participants, their data, or biological material

Policy information about studies with human participants or human data. See also policy information about sex, gender (identity/presentation), and sexual orientation and race, ethnicity and racism.

Reporting on sex and gender	Study design considered self-reported sex, and data were analyzed and results are reported by sex
Reporting on race, ethnicity, or other socially relevant groupings	No socially constructed/relevant variables were used
Population characteristics	Data were from a large population-based cancer registry data of the United States called Surveillance, Epidemiology, and End Results.
Recruitment	No recruitment was performed because the data were publicly available.
Ethics oversight	Surveillance, Epidemiology, and End Results Cancer Registry provides data publicly and our manuscript used only the publicly available data

Note that full information on the approval of the study protocol must also be provided in the manuscript.

Field-specific reporting

Please select the one below that is the best fit for your research. If you are not sure, read the appropriate sections before making your selection.

☒ Life sciences ☐ Behavioural & social sciences ☐ Ecological, evolutionary & environmental sciences

For a reference copy of the document with all sections, see nature.com/documents/nr-reporting-summary-flat.pdf

Life sciences study design

All studies must disclose on these points even when the disclosure is negative.

Sample size	Surveillance, Epidemiology, and End Results Cancer Registry provides data publicly and our manuscript used their publicly available data. Thus, there was no sample size determination.
Data exclusions	No data were excluded from the publicly available data Surveillance, Epidemiology, and End Results Cancer Registry provided.
Replication	Replication of the applicability of the cancer incidence model was performed using different cancer types of Surveillance, Epidemiology, and End Results Cancer Registry
Randomization	No randomization was used: this was not a trial.
Blinding	No group allocation was used: this was not a trial.

Reporting for specific materials, systems and methods

We require information from authors about some types of materials, experimental systems and methods used in many studies. Here, indicate whether each material, system or method listed is relevant to your study. If you are not sure if a list item applies to your research, read the appropriate section before selecting a response.

Materials & experimental systems

n/a	Involved in the study	
☒	☐	Antibodies
☒	☐	Eukaryotic cell lines
☒	☐	Palaeontology and archaeology
☒	☐	Animals and other organisms
☒	☐	Clinical data
☒	☐	Dual use research of concern
☒	☐	Plants

Methods

n/a	Involved in the study	
☒	☐	ChIP-seq
☒	☐	Flow cytometry
☒	☐	MRI-based neuroimaging

Plants

Seed stocks	N/A
Novel plant genotypes	N/A
Authentication	N/A
